# Evaluation and comparison of tools used to quantify aggregate PFAS exposure: Extractable organic fluorine, PFAS burden scores and summed PFAS concentrations

**DOI:** 10.1038/s41370-025-00806-x

**Published:** 2025-10-02

**Authors:** Rachel A. Klein, Shelley H. Liu, Joseph M. Braun, Katherine E. Manz

**Affiliations:** 1 Department of Environmental Health Science, University of Michigan, 1415 E Washington Heights, Ann Arbor, MI 48109 USA; 2 Department of Population Health Science and Policy, Icahn School of Medicine at Mount Sinai, New York, New York, 10029 USA; 3 Department of Epidemiology, Brown University, Providence, Rhode ISL 02912 USA

**Keywords:** Extractable organic fluorine, Targeted PFAS analysis, PFAS burden scores, Exposure assessment

## Abstract

**Background:**

Bioaccumulation, widespread usage, and adverse human health effects emphasize per- and polyfluoroalkyl substances (PFAS) as an important public health concern. There is a need for an aggregate PFAS exposure measure due to the increasing diversity of structures. Aggregate measures are important for informing clinical care, biomonitoring, and research standardization. Current approaches for human biomonitoring of PFAS include targeting and quantifying a limited number of molecules and estimating exposure based on summed concentrations or statistical modeling. Extractable organofluorine (EOF) has been proposed as an aggregate PFAS biomarker that quantifies the total organically bound fluorine in a sample, encompassing PFAS regardless of knowing the exact chemical structures. However, EOF in human biomonitoring studies or environmental epidemiology is limited.

**Objective:**

The objective of this study is to comprehensively assess human studies that measure EOF and target PFAS in the same sample by conducting a literature search, data extraction, and secondary data analysis.

**Methods:**

We assessed the correlation of three aggregate PFAS exposure metrics with each other: EOF, adjusted summed concentrations of PFAS identified by the National Academies of Science Engineering and Medicine (NASEM), and PFAS burden scores.

**Results:**

Across 8 published studies from US, Asia and Europe with 163 samples, EOF concentrations were higher than NASEM summed PFAS concentrations, and EOF was strongly associated with PFAS burden scores and NASEM sum. EOF does not identify or differentiate non-PFAS sources of fluorine which limits identification of individual molecules and their potential toxicity.

**Significance:**

Correlations between EOF, summed targeted PFAS concentrations, and PFAS burden scores demonstrated that EOF is a practical tool for estimating PFAS exposure and identifying individuals with high exposure to PFAS. Thus, EOF could be utilized for identifying individuals or sub-populations with high aggregate PFAS exposure. Practical considerations in laboratory analyses, including instrumentation, sample matrix, and sample extraction procedure, remain potential barriers to widespread implementation of EOF as a biomonitoring tool.

**Impact:**

- This study highlights the potential of extractable organofluorine (EOF) as a comprehensive biomarker for assessing aggregate PFAS exposure in human populations. By analyzing data from eight studies across the US, Asia, and Europe, we found that EOF concentrations were higher than summed PFAS concentrations and correlated strongly with PFAS burden scores. Although EOF does not identify specific fluorine sources, its strong associations suggest it is a practical tool for detecting high PFAS exposure. While EOF offers promise for identifying at-risk populations, practical challenges in laboratory analyses may limit its widespread use in biomonitoring programs.

## Introduction

Per- and polyfluoroalkyl substances (PFAS) are a class of persistent chemicals [1] used in manufacturing and production for a variety of consumer and industrial products [2]. While PFAS definitions vary [3], they are characterized by their composition of carbon-fluorine bonds. These bonds contribute to PFAS characteristics such as resistance to degradation and biotransformation [4], as well as longevity in the environment and biological organisms [5]. The first definition of PFAS was published in 2011 by Buck et al., which defined PFAS as carbon chains containing 1 or more carbon atoms (C) bonded together, where the available bonding sites are occupied by at least two fluorine (F) atoms, represented by C_n_F_2n+1_ [6]. The US Environmental Protection Agency (EPA) defined PFAS most recently via the Toxic Substances Control Act (TSCA) as chemicals containing at least one of the following structures: “R-(CF_2_)-CF(R′)R″, where both the CF_2_ and CF moieties are saturated carbons; R–CF_2_OCF_2_-R′, where R and R′ can either be fluorine (F), oxygen, or saturated carbons (C); or CF_3_C(CF_3_)R′R″, where R′ and R″ can either be F or saturated carbons” [7]. The Organization for Economic Cooperation and Development (OECD) define PFAS as a fully or partially fluorinated C with at least 2 F atoms bonded to 1 or more C atoms [8]. More recently, PFAS have been classified based on their substructure and total composition of fluorine atoms, requiring at least 30% of the total atoms to be fluorine [9]. The evolving definitions of PFAS have made it difficult to define and characterize chemicals [10], complicating risk assessment and investigations into associated health outcomes. Efforts to regulate PFAS as a chemical class are complicated by unclear and changing definitions, contributing to contentious debates for industry and health [10]. For example, a concern of PFAS class-wide regulation is that there is no evidence or guarantee that the replacement chemicals are safer, or less toxic for human health [11].

PFAS have been investigated and associated with adverse health outcomes in humans and animals [12], highlighting their critical importance in the field of environmental health, including primary prevention of disease through identifying highly exposed populations for proactive monitoring and intervention [13, 14]. Health effects of individual PFAS include immunosuppression and an altered immune response [12, 15, 16], tetanus [17], and measles [18], reduced antibody response to vaccination [19], endocrine disruption in women, (demonstrated by effects on hormone production, menstruation, and fertility) [20], hormone production and infertility in men [21], metabolic alterations [22–24], gestational weight gain [25], and reduced birth weight [26, 27]. Individual PFAS have been associated with increased rates of cancer in human studies, including strong evidence for renal [28] and testicular cancer [29], and moderate evidence for breast [30] and thyroid cancer [31].

Quantifying individual PFAS chemicals is crucial for assessing specific disease risks and implementing appropriate interventions [32]; however, the increasing diversity of PFAS structures released into the environment [33] complicates the assessment of overall PFAS exposure. Quantifying aggregate health effects of PFAS exposure is critical given that humans are exposed to multiple PFAS simultaneously [34] (i.e., PFAS mixture). PFAS are diverse in structure and usage [35]; consequently, the molecules humans are exposed to can also vary widely. In the early 2000’s, PFAS manufacturers began voluntary phase out [36], or elimination from production in the United States and Europe, of two common PFAS, perfluorooctanoic acid (PFOA) and perfluorosulfonate (PFOS), which are now referred to now as legacy PFAS [2]. With the reduction in production of these PFAS, new and emerging chemicals are created and substituted where PFOS and PFOA were once used [37]. The number of PFAS are increasing rapidly [38]; based on the OECD definition of PFAS, there may be over 7 million unique PFAS [39]. There are approximately 1.7 million unique chemical structures currently discoverable via Google Patents that fit at least 1 of 3 PFAS defintions [40].

Currently, PFAS testing in humans is most commonly carried out via targeted analysis of a limited number of known PFAS [41] which have analytical standards commercially available [42]. These compounds are selected based on known toxicity and detectable levels in human samples [41]. For example, the Centers for Disease Control and Prevention currently provides biomonitoring estimates for 8 unique PFAS in the serum of the US population [41]. Non-targeted analysis (NTA) has been used to characterize PFAS exposures that may not have been suspected or for structures that do not have analytical standards commercially available [43, 44]. However, NTA does not currently provide absolute concentrations. The diverse mixture of possible chemical exposures makes estimating aggregate PFAS exposure complex and emphasizes the necessity for a comprehensive or aggregate PFAS measurement [45].

### Aggregate PFAS metrics: NASEM sum, burden scores, and extractable organic fluorine

Aggregate approaches for determining PFAS exposure include summing concentrations of measured PFAS compounds, PFAS burden scores derived using item response theory that are based on U.S. population exposure reference ranges, and total fluorine concentrations (Table 1). Summed approaches for estimating total exposure add concentrations of PFAS targeted in each study. One example of a summed approach was released by the National Academies of Sciences, Engineering, and Medicine (NASEM) in their Guidance on PFAS Exposure, Testing, and Clinical Follow-Up Report [46]. The NASEM recommendations were based on German Human Biomonitoring (HBM) values for PFOA and PFOS that resulted in adverse health effects, and an additional 5 molecules: Methylperfluorooctane sulfonamidoacetic acid (MeFOSAA), Perfluorohexanesulfonic acid (PFHxS), Perfluorodecanoic acid (PFDA), Perfluoroundecanoic acid (PFUnDA), and Perfluorononanoic acid (PFNA). Based on the PFOA and PFOS HBM values, the NASEM Guidelines recommended that the sum concentrations of serum or plasma be used to inform clinical care by the following standards: < 2 ng/ml is not expected to have adverse health effects, 2–20 ng/mL poses potential for adverse health effects especially for vulnerable populations, and >20 ng/L poses an increased risk of health effects [46].

**Table 1:** Comparison of aggregate PFAS measures.

Metric	Goal/Intended Use	Measurement/Calculation	Strengths	Limitations
NASEM Sum	Expert scientific recommendations for “Guidance on PFAS Exposure, Testing, and Clinical Follow-Up” from the National Academies of Sciences, Engineering, and Medicine (NASEM) in 2022 [46]	• Targeted LC-MS/MS analysis of 7 molecules • Additive sum of measured concentrations • Molecules: PFOS, PFOA, MeFOSAA, PFHxS, PFNA, PFDA, PFUnDA	• Connected to clinical guidance with thresholds for additional clinical monitoring • Straightforward comparison between studies where targeted data was collected • Necessitates studies to measure all 7 PFAS molecules	• Need for analytical reference standards for each chemical • Differing toxicity of individual PFAS
PFAS Burden Scores	Summary metric score for PFAS burden using the 2017-2018 US PFAS Exposure Burden Calculator, which was calibrated to nationally representative PFAS biomonitoring data and constructed using item response theory (IRT) [47]	• Targeted LC-MS/MS analysis of 7 molecules • Concentrations uploaded to burden calculator online tool • Molecules: PFOS, PFOA, MeFOSAA, PFHxS, PFNA, PFDA, PFUnDA	• Burden scores can be interpreted relative to the general US population • Allows cross-study comparisons on a common scale, even if studies do not measure all 7 PFAS molecules	• Need for analytical reference standards for each chemical
Extractable Organic Fluorine (EOF)	Total amount of extractable organically bound fluorine in a sample that provides an aggerate measure of all organofluorine compounds able to be extracted given the extraction conditions [51]	• Combustion Ion-Chromatography	• No need for analytical reference standards of individual chemicals [74] • High inclusivity, including PFAS that may not have analytical standards [74] • Simplified measurement for risk communication	• No information on specific chemical structures or concentrations • Differing toxicity levels of individual PFAS • Extraction procedures have not been standardization [74] • Limited information to develop targeted interventions

Summary metric scores are statistical tools that summarize burden for a chemical class, independent of the health outcome. For PFAS, Liu et al. used item-response theory (IRT) to develop a PFAS burden calculator, calibrated on nationally representative US PFAS biomonitoring data [47], that outputs a burden score based on the blood concentrations of specific PFAS (Calculator Available at: https://pfasburden.shinyapps.io/app_pfas_burden/) [48]. The tool was designed to create a standardized measure of cumulative PFAS exposure that could be easily compared across studies and facilitate cross-study harmonization. It enables researchers to calculate PFAS exposure burden scores on a common scale, so that PFAS burden estimates can be compared across studies, even if researchers do not measure exactly the same set of PFAS analytes [47]. Using a sum-score would require that the studies measure a common set of analytes. The PFAS burden calculator uses survey-weighted (using PFAS subsample weights) decile cutoffs for categorizing each PFAS analyte. The chemicals included in the PFAS Burden calculator are: 2-N-methyperfluorooctanesuldonamido (me-PFOSA-AcOH), PFUnDA, PFDA, PFHxS, PFNA, PFOA, and PFOS [47]. Benefits of this tool are that the burden score considers patterns of concentrations (not the summed concentrations) and that it has been calibrated using biomonitoring data from the CDC’s National Health and Nutrition Examination Survey (NHANES) [49] for PFAS collected between 2017 and 2018. The PFAS burden score is on a standard normal scale (mean 0, standard deviation 1). A score of 0 indicates an individual has average PFAS burden as compared to the general US population from 2017–2018, while a score of 1 means a person has PFAS burden that is one standard deviation above average, and a score of −1 means a person has PFAS burden one standard deviation below average. In general, a negative PFAS exposure burden means the participant’s PFAS exposure is below average, based on population-level reference ranges from 2017 to 2018. This allows for subtle differences in exposure burden that may not be observed with summed concentrations, which may also be sensitive to outliers’ [47]. Based on our previous work [47, 50], we have shown that Item-Response-Theory derived scores were able to detect associations with health outcomes that were not detected or had smaller effect sizes when using summary scores as the summary metric.

Finally, total fluorine (TF) analysis is analytical chemistry tool that quantifies the total amount of organic and inorganic fluorine in a sample using combustion ion chromatography (CIC) [51]. While TF includes both the organic and inorganic fractions, the organic fraction can be extracted from a sample, extractable organic fluorine (EOF) [52]. EOF includes the soluble, organically bound fluorine in a sample, and is an aggregate measure of all organofluorine compounds within the sample [53]. Thus, EOF provides an aggregate measure of organofluorine concentrations including PFAS with known structures and available standards, as well as those that do not have standards [51]. EOF has also been combined with targeted PFAS analysis to quantify the amount of identified and unidentified fluorine levels in human samples (often referred to as the “Fluorine Mass Balance Approach”) [52, 54]. In the fluorine mass balance approach, the concentration of each targeted PFAS is converted to equivalents of fluorine and subtracted from the total amount of fluorine by EOF, the remaining fluorine is often referred to as unidentified and/or unsuspected PFAS [51]. EOF is a useful approach in that it provides a total measure of organofluorine without needing to know and target exact chemical structures, which is especially useful as replacement PFAS are developed at a faster rate than standards are becoming available [32].

The increasing number of PFAS structures and lack of commercially available analytical standards [38, 41], emphasize the need for and importance of a total exposure measurement for assessing PFAS. Given that summing concentrations of known PFAS tends to focus on legacy chemicals, other PFAS exposure may be overlooked. We compared these three measures for aggregate PFAS exposure (summed concentrations using the NASEM-identified PFAS, PFAS burden scores, and EOF). We did so through a literature review, data extraction, and quantitative secondary data analysis of studies that have measured EOF and targeted PFAS in human blood and calculated summed concentrations and the PFAS burden scores.

## Methods

### Literature review and data extraction

We searched peer-reviewed literature to identify studies that performed targeted analysis of the NASEM recommended PFAS and extractable organofluorine analysis on the same sample. We used search engines to identify relevant research in PubChem, PubMed and Google Scholar. We utilized the Abstract Sifter Excel tool to query PubMed [55]. The following four searches were made and the resulting articles were individually evaluated for adherence to our inclusion criteria after each search: “PFAS and EOF and HUMAN”, “PFCs and EOF and HUMAN”, “PFAS and FLUORINE and HUMAN”, and “PFCs and FLUORINE and HUMAN”. Our search began in January of 2024 and was repeated for new literature monthly until June 2024.

For the purposes of this study, we identified peer-reviewed studies that fit the following inclusion criteria: human plasma or serum sample, performed targeted PFAS analysis, and performed extractable organofluorine analysis via combustion ion chromatography. We did not limit the extraction techniques used for sample preparation for chemical analyses. For targeted PFAS analysis, we included studies that analyzed at least 6 of the 7 NASEM recommended PFAS. We included data from individual or pooled samples, and both pooled and individual samples were included in our comparison.

Our inclusion criteria resulted in 8 peer-reviewed research studies that we extracted the data from. We extracted descriptive characteristics and demographics including year sample collected, sample location, and exposure origin. We also noted when the data represented individual samples or pooled samples. We then classified data into two categories of PFAS exposure based on their original published research: samples were noted as general population if there were no known sources of elevated PFAS exposure, or as high exposure (“highly exposed”) from occupational and high known exposures ( >80 ng F/ml).

### Data management

Extractable Organofluorine: EOF was reported in concentrations of ng F/ml in all studies. Samples with EOF values below LOD were not included in this study because the data from the original studies were excluded if below the LOD [56]. The extraction methods from each study are noted in Table 2.

**Table 2:** Identified Study Information. Median and IQR results for EOF, NASEM Sum, Unidentified EOF (UOF), and PFAS Bun Scores.

Key	Sample Description	Pooled or Individual	Sample *N*	Study Location	Sex/gender	Date(s) Sample(s) Collected	EOF Extraction*		EOF (ng F/ml) Median, 25–75%	PFAS Σ (ng F/ml) Median, 25–75%	Non-NASEM F (ng/ml F) Median, 25–75%		PFAS Burden (0 = NHANES average) Median, 25–75%
A	General population samples for comparison	Individual	9	Sweden	44.4% F	2014-2015	Ion-pair (TBA) & LLE w/ MTBE		20.9 12.1-29.7	4.08 0.394-7.77	17.4 9.78-25.0		-0.0401 -0.394-0.314
A	Exposed samples: AFFF contaminated drinking water	Individual	20	Sweden	65% F	2014-2016	Ion-pair (TBA) & LLE w/ MTBE		222 96-348	209 58.1-359	49.2 -69.8-168		0.884 0.248-1.52
B	General population samples, pooled by age and gender	Pooled	130 (6 pools)	Sweden	52% F	2018	Ion-pair (TBA) & LLE w/ MTBE		8.80 4.12-13.5	3.37 2.39-4.34	4.39 -1.48		-0.0727 -0.143-(-0.0024)
C	Exploration of patient cohort investigating type 2 diabetes	Pooled	472 (3 pools)	Norway	52-57% F	1986, 2007, 2015	LLE with acetonitrile		20.5 18.1-23.0	12.7 9.18-16.3	5.65 0.682-10.6		1.42 0.649-2.19
D	Investigation of maternal and cord specimens	Pooled	82 (32 pools)	Austria	100% F maternal, 50% F cord	2018	SPE-WAX		2.1 .91-3.29	1.84 0.582-3.10	0.543 -0.175-1.26		-0.702 -1.29-(-0.116)
E	General population samples used as control group	Individual	7	USA/Japan	100% M	2001, 2003	Ion-pair (TBA) & LLE w/ MTBE		45.1 17.5-72.7	39.6 11.8-67.4	3.38 2.07-4.69		1.73 0.17-3.29
E	Occupational exposure workers in fluorotelomer manufacturing	Individual	4	Japan	100% M	2004	Ion-pair (TBA) & LLE w/ MTBE		762 217-1310	749 129-1370	-11.9 -49.2-25.4		2.41 2.32-2.51
F	Exploration of volunteers in universities/hospitals	Pooled	30 (5 pools)	China	50% F	2004	Ion-pair (TBA) & LLE w/ MTBE		21.0 9.1-32.9	12.2 0.390-23.7	6.57 0.763-12.4		0.0403 -0.374-0.454
G	Case control investigations of impacts of pharmaceutical usage	Individual	20	USA	50% F	2020-2021	Ion-pair (TBA) & LLE w/ MTBE		6.26 3.38-9.13	3.58 0.557-6.60	1.08 -1.81-3.96		-0.0456 -0.687-0.596
H	Investigation of first-time mother from the general population.	Pooled	472 (57 pools)	Sweden	100% F	1996-2017	LLE with acetonitrile	35.6 23.1-48.1		16.1 9.73-22.5		12.9 9.19-34.7	0.998 0.534,1.462

*TBA Thiobarbituric Acid, *LLE* Liquid-Liquid Extraction, *MTBE* Methyl tertiary-butyl ether, *SPE* Solid Phase Extraction, *WAX* Weak Anion Exchange.

Targeted PFAS: Targeted PFAS analysis results were reported in ng/ml or ng/g. The targeted concentrations reported in ng/g were converted to ng/ml by multiplying samples by the density of blood serum as 1.02 g/ml [57]. We recorded the associated LOD and/or LOQ with associated units for each targeted chemical in each study and replaced targeted PFAS results below LOD with LOD/√2. MeFOSAA concentrations were imputed as 0 for studies that did not conduct targeted analysis of the chemical prior to further analysis.

Fluorine-Adjusted NASEM sum for Fluorine Mass Balance Analysis and EOF Comparison: The fluorine mass balance (FMB) approach was used to estimate the amount of EOF explained and unexplained by the measured targeted PFAS concentrations. First, targeted PFAS concentrations were converted to equivalents of fluorine per molecule for comparison to EOF using the following equation:

1 $$\sum F={C}_{i}\times n\times \frac{{{MW}}_{{Fluorine}}}{{{MW}}_{i}}$$

Where Ci is the concentration of the i-th targeted PFAS, n is the number of fluorine atoms per molecule of the i-th PFAS, MW_Fluorine_ represents the molecular mass of fluorine as each PFAS (MW_i_), and summaF is the resulting concentration of the molecule in equivalents of fluorine. The sum concentration (referred to as “Fluorine-Adjusted NASEM Sum” from here on) was then calculated by summing the concentrations of the seven PFAS in Table 1 after conversion to equivalent of fluorine (ng F/ml). The following equation was used for fluorine mass balance analysis to quantify the percent of EOF that could not be identified by the Fluorine-Adjusted NASEM Sum, where EOF and the Fluorine-Adjusted NASEM Sum both have ng F/mL units, which will be referred to as non-NASEM Fluorine.

2 $$\% {non}-{NASEM}F=\frac{({EOF}-{Fluorine\; Adjusted\; NASEM\; Sum})}{{EOF}}\times 100 \%$$

We adjusted the HBM cutoff levels adopted by NASEM to evaluate EOF and targeted PFAS concentrations in fluorine equivalents based on the clinical recommendation. We used Eq. 1 to convert the recommended concentrations cutoffs to equivalents of fluorine atoms. We used PFOA since it is the molecule contributing the most to the NASEM sum. The cutoffs values of 2 and 20 ng/ml were calculated as 1.3 and 13 ng F/ml.

PFAS Burden Scores: We previously implemented IRT to calibrate the 2017-2018 US PFAS exposure burden calculator, which estimates an individual’s PFAS burden score as a method to capture the totality of exposure to PFAS mixtures [48]. This PFAS burden calculator was calibrated using 2017-2018 NHANES data from the general US population. In brief, the IRT approach relates PFAS biomarker concentrations to a latent PFAS burden score via nonlinear functions. To avoid undue influence of outliers, and because IRT methods are primarily for categorical data, the 2017–2018 PFAS burden calculator uses discretized PFAS data. Continuous PFAS biomarker concentrations are discretized into ordinal data using survey-weighted quantile cutoffs from 2017–2018 NHANES, with up to 10 categories per PFAS biomarker. A strength of IRT is that it allows for the inclusion of mixed item types without the need for imputation (e.g., using deciles for frequently detected chemicals, and quartiles for less frequently detected chemicals). As the PFAS burden calculator was previously calibrated and validated, is publicly available, other studies can input PFAS biomarker data into our calculator, to estimate PFAS burden scores on the same scale which can be interpreted relative to the general US population. The PFAS burden scores are on a standard normal scale (mean 0 - indicating that a person has average PFAS burden for the population - and standard deviation 1 – indicating that a person has PFAS burden one standard deviation below or above average). A key strength of the PFAS burden calculator is that it enables a common scale, such that PFAS burden scores can be compared across studies even if they do not measure the exact same set of PFAS.

The extracted PFAS concentrations of the 7 molecules, PFNA, PFDA, PFUnDA, PFOA, PFOS, PFHxS, and MeFOSAA (Table 1) were uploaded to the PFAS exposure burden calculator in ng PFAS/mL units and burden scores were calculated for each sample or pool (https://pfasburden.shinyapps.io/app_pfas_burden/).

### Correlation analyses

We conducted all analyses using R versions 4.3.0. We conducted multiple spearman correlation analyses to assess the strength of relationship between EOF, NASEM sum, and PFAS burden. First, we compared Fluorine-Adjusted NASEM sum and EOF. We also compared PFAS burden to Fluorine-Adjusted NASEM sum and EOF.

## Results

Our literature search resulted in between 3 and 231 studies. Out of these studies, 8 were selected based on their adherence to the inclusion criteria (Table 2) [54, 56, 58–63]. Excluded studies included research conducted on water, dust, and other textiles as well as studies that did not measure at least 6 of the targeted molecules outlined by NASEM. Five of 8 studies collected targeted data on all the NASEM molecules, 3 studies measured 6 of the 7 molecules, these 3 did not measure MeFOSAA. Samples were reported from the USA, Sweden, Norway, Japan, China, and Austria (Table 2). Across all studies, samples were collected between the years 1986 and 2021 (Table 2). We identified 160 samples across the 8 studies, 69 individual samples in 4 of the studies and 9 pooled samples in the other 4 studies. A total of 102 samples reported EOF values above the LOD. In the studies with pooled samples, the samples were combined based on demographic information, exposure status, and/or collection methods (Table 2).

We categorized the samples into two groups based on known PFAS exposure in the original research: (1) the general population, which included volunteers, controls, and patient cohorts that did not have high known exposure to PFAS, and (2) highly exposed, which included samples with specific sources identified as high PFAS exposure ( > 80 ng F/ml). Samples with known high exposure were from communities with known AFFF water contamination from firefighting foams in Sweden (Aro et al.) and from occupational exposed workers in fluoropolymer manufacturing in Japan (Miyake). All samples in the general population were collected as control groups for the two highly exposed populations, or from samples collected from the general population.

**Fig. 1:**
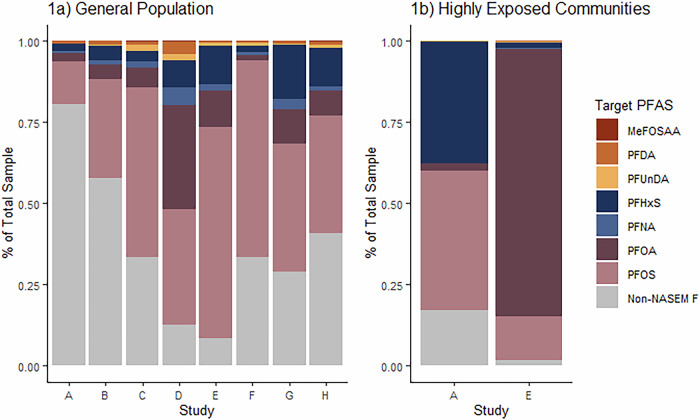
The fluorine mass balance results for the 102 samples considered from 8 studies with targeted PFAS results and EOF values above LOD. Samples are divided by the general population (left panel, 1**a**) and those with high known exposures (right panel, 1**b**). Non-NASEM F refers to fluorine identified via EOF analysis that could not be attributed any of the 6–7 targeted analytes tested in each study. The study legend can be found in Table 2. Percentages of detected PFAS and non-NASEM fluorine (ng F/ml) and can be found in Supplemental Table 1a for the general population and Supplemental Table 1b for highly exposed samples.

The fluorine mass balance results illustrate the aggregate PFAS concentration for each study using the portions of EOF described by the Fluorine-Adjusted NASEM Sum and those that are not accounted for in this approach (Non-NASEM F) (Fig. 1). On average, samples from the general population consisted of 40% (13-90%) organic fluorine not described by the Fluorine-Adjusted NASEM Sum (Supplemental Table 1a). However, this ranged from a minimum of 13% of Study E to >50% in studies, A, B, and H (Fig. 1a, Supplemental Table 1a).

For the highly exposed samples, their non-NASEM fluorine concentrations were <1–8% on average, indicating that most of their PFAS body burden was derived from a small number of known individual PFAS molecules (Supplemental Table 1b). For the highly exposed samples, PFOS concentrations were highest in the study where participants were exposed to AFFF-contaminated drinking water in Sweden, and PFOA was the main source of occupational exposure for the fluoropolymer manufacturing workers in Japan (Fig. 1b).

**Fig. 2:**
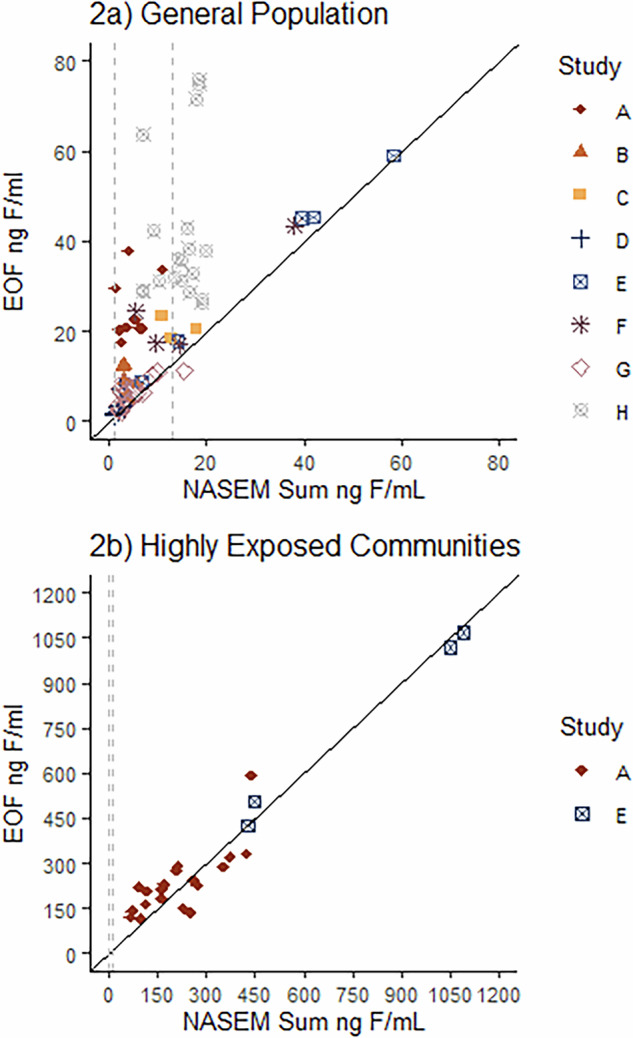
EOF concentrations plotted in relation to the NASEM sum. EOF and NASEM sum are reported in nanograms of fluorine per milliliter. 2**a** includes samples collected from the general population. 2**b** represents samples from studies with known high exposures, including community and occupational exposures. Average concentrations of Fluorine-Adjusted NASEM sum and EOF in ng F/ml can be found in Supplemental Tables 2a and 2b. The solid black line represents a 1:1 ratio of EOF and NASEM (slope of 1). The grey dashed lines represent the NASEM sum clinical care recommended ranges converted to ng F/ml assuming the entire exposure is from PFOS using equation (1). PFOS was used as an estimate of exposure since it was the most common main exposure for the samples in our study. The study legend is in Table 2. Spearman correlation results: 2a, *r* = 0.811, *p* < 0.0001, *N* = 79; 2**b**, *r* = 0.858, *p* < 0.0001, *N* = 23 (Supplemental Table 2c).

EOF and Fluorine-Adjusted NASEM Sum concentrations in equivalents of fluorine were compared to assess their agreement with each other as there is no gold standard to assess aggregate PFAS exposure (Fig. 2a, b). On average, EOF was higher than Fluorine-Adjusted NASEM sum in the general population and highly exposed samples (Supplemental Tables 2a and 2b). In all but 3 of the 139 samples reported from the general population, EOF provided higher estimations of total organic fluorine concentrations than the Fluorine-Adjusted NASEM Sum (Fig. 2a). Surprisingly, EOF underestimated fluorine compared to the Fluorine-Adjusted NASEM concentrations in 3 samples by 0.67, 0.44, and 4.20 ng F/ml, each of these samples all originated from the same study (Miyake et al., Study G). The relationship between EOF and Fluorine-Adjusted NASEM sum in the general population was strong, spearman’s *r* = 0.811, *p* < 0.0001 (*N* = 79) (Fig. 2a, Supplemental Table 2c). It is an unexpected result that Fluorine-Adjusted NASEM sum concentrations of fluorine would be higher than EOF values since EOF aims to encompass all sources of fluorine. These differences can be potentially attributed to experimental error (11-37%) and sample extraction techniques (discussed in more detail below).

For the highly exposed samples, EOF was lower than the NASEM Sum in 10 of the 23 samples, 7 from Study A and 3 from Study E (Fig. 2a). At high ( > 80 ng F/mL) concentrations, the relationship between fluorine from EOF and fluorine from the Fluorine-Adjusted NASEM sum was strongly correlated (Spearman’s *r* = 0.858, *p* < 0.0001, *N* = 23) (Fig. 2a, Supplemental Table 2c). EOF more frequently estimated higher concentrations of fluorine compared to the Fluorine-Adjusted NASEM sum. We can attribute this difference to additional sources of organofluorine, including additional PFAS, not included in the Fluorine-Adjusted NASEM sum.

We utilized the converted HBM values used in the NASEM Guidelines to fluorine equivalents. Of the 102 samples with EOF measurements across all studies, 2 samples were below the value 1.3 ng F/mL (not expected to have adverse health effects), 38 samples were in the range 1.3-13 ng F/mL (poses potential for adverse health effects especially for vulnerable populations), and 62 samples were above the higher limit of concern >13 ng F/mL (poses an increased risk of health effects) (Fig. 2a). Of the 62 samples we identified with concentrations above the upper HBM limits at risk of adverse health effects, 38 of these samples were control or general population samples without known sources of PFAS exposure. All the samples from the two studies that focused on highly exposed communities fell into the >13 ng/mL range, indicating their high risk for adverse health effects.

**Fig. 3:**
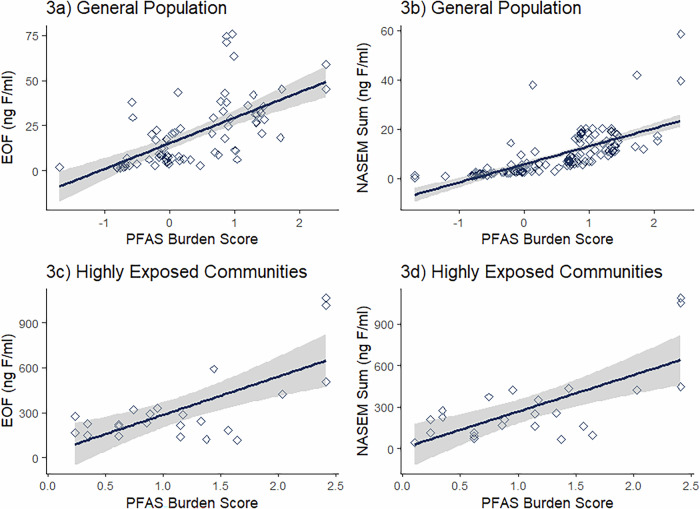
EOF and NASEM Sum concentrations plotted in relations to burden scores. 3**a** and 3**b** includes samples collected from the general population. 3**c** and 3**d** represents samples from studies with known high exposures, including community and occupational exposures’ burden scores are standardized against a national sample, with a score of 0 indicating no difference in PFAS burden compared to the reference population Spearman correlation results: 3**a**, *r* = 0.721, *p* < 0.0001, *N* = 79; 3**b**, *r* = 0.883, *p* < 0.0001, *N* = 139; 3**c**, *r* = 0.403, *p* = 0.053, *N* = 23; 3**d**, *r* = 0.541, *p* = 0.006, *N* = 24 (Supplemental Table 3).

We also compared both EOF and Fluorine-Adjusted NASEM to the PFAS burden scores (Fig. 3). EOF was strongly associated with burden scores in the general population and moderately associated in highly exposed communities, *r* = 0.721 (*p* < 0.0001, *N* = 79) and *r* = 0.403 (*p* = 0.053, *N* = 139), respectively (Fig. 3a and Fig. 3c, Supplemental Table 3). Fluorine-Adjusted NASEM sum concentrations were strongly associated with burden scores in the general population (*r* = 0.883, *p* < 0.0001, *N* = 23), and moderately associated in highly exposed communities (*r* = 0.541 *p* = 0.006, *N* = 24) (Fig. 3b and d, Supplemental Table 3).

## Discussion

We acknowledge that there are several limitations to using EOF as an aggregate PFAS exposure assessment measurement, which require additional research. The first limitation resides in the constraints of the analytical technique, including the instrument sensitivity and extraction methods [64]. Variability in analytical techniques, instrumentation, and sample preparation methods can explain the differences in fluorine detected in EOF and NASEM sum. One paper evaluated three different extraction techniques used in extractable organofluorine analysis, ion-pair extraction followed by liquid-liquid extraction, and solid-phase extraction using hydrophilic-lipophilic balance (HLB) or weak ion exchange (WAX) techniques [64]. The study found that EOF concentrations vary based on extraction technique, for example, SPE-HLB resulted in the highest overall concentrations of fluorine, but also exhibited the greatest variability between samples and SPE-WAX had the least amount of variability [64]. The differences in EOF values between techniques indicate differences in the efficiency of various methods and highlight a limitation of comparing EOF values between extraction techniques. EOF analysis was conducted with starting sample sizes between 2 mL and 100 uL for the identified studies [56, 58–61], compared to Environmental Protection Agency method 1633 which provides guidance for PFAS testing of aqueous samples based on a starting sample sized of 500 mL [65].

Second, EOF is limited in that it does not identify the unique chemicals contributing to total PFAS burden, thus the origins of the chemicals are unknown. Individual chemical quantitation and identification may be important for identifying exposure sources [34], thus EOF may not be the ideal measure for discovery-based investigations. However, EOF may be beneficial for general exposure assessment and identification of highly exposed people [58], especially for chemicals which analytical standard availability is limited. The NASEM clinical guidelines acknowledge the potential organofluorine measurements to measure vast and diverse molecules (Box 5-1) [46]. A potential source of bias may be fluorinated pharmaceuticals [66], which may be a possible source of non-PFAS fluorine in human samples. Pennoyer et al., investigated pharmaceutical usage via patient self-reported surveys in addition to 44 targeted PFAS and EOF concentration in blood and found non-significant differences in EOF among users and non-users of fluorinated pharmaceuticals (difference: 0.36 ng F/ml; 95% CI: -1.26 to 1.97 ng F/ml). However, given the small sample size (*n* = 10), there is not strong evidence to conclude if, and by how much, pharmaceutical usage impacts blood organic fluorine concentrations based on this study. A recent analysis (same cohort at study C) included fluorinated pharmaceutical in their fluorine mass balance evaluation of human samples [67]. This study found that the proportion of EOF that accounted for pharmaceuticals increased over time on average, from 0% in 1986, to 6.4% in 2007, and 39% in 2015 [67]. In 2020, 20% of globally registered pharmaceuticals contained fluorine [63]. Overall, pharmaceuticals are thought to have short half-lives and low propensity to bioaccumulate [68]. Despite this evidence, it is possible that pharmaceuticals remain present in the blood due to frequency of consumption and may contribute to organic fluorine levels. Pesticides are another potential additional source of fluorine exposure [69]. Further research should investigate if, and by how much, pesticide exposure contributes to total fluorine exposure. The half-life of specific PFAS may also influence the EOF measured in a sample. PFAS with shorter half-lives may exhibit more within-person variability; therefore, PFAS with shorter half-lives may not be as well represented in EOF. This may explain the observed greater correlation between EOF and targeted PFAS in the community highly exposed to the persistent source than in the general population where less persistent PFAS likely make up a greater proportion of the EOF. Therefore, future research may consider how effectively short-chain or non-persistent PFAS are captured in EOF measurements.

Finally, while EOF provides a measure of total PFAS exposure, it does not necessarily translate directly to total toxicity or health effects experienced by individual humans. Different PFAS can have distinct impacts on different biological systems, such as the endocrine [21], metabolic [24], and neurological systems [27]. While individual PFAS may have distinct biological effects, future research will need to determine whether EOF is related to specific outcomes across populations. Moreover, not knowing the specific types of PFAS within the EOF mixture will not inform how to reduce or prevent exposures. Therefore, a multi-method approach could be taken: EOF can provide an overall indication of total exposure burden, while detailed analyses of individual PFAS can inform exposure and specific health effects. In scenarios where resources are limited, prioritization becomes key. One potential approach is to use EOF as an initial screening tool to identify populations or individuals with high exposure levels. This can guide where more detailed investigations and targeted interventions are most needed. Ultimately, the choice of prioritization may depend on the specific context, including the availability of resources, the known prevalence of certain PFAS, and the specific health outcomes of concern. However, we acknowledge that in some cases, such as when elevated EOF levels are detected in drinking water, a non-specific intervention like water filtration could be recommended without detailed quantitation of individual PFAS. While using EOF has some limitations with regard to identifying specific sources of exposure or constituent PFAS with the most toxic effects, the use of such an aggregate measure is not without precedent. The Environmental Protection Agency regulates total trihalomethanes (TTHM), which is a mixture of four volatile organic compounds formed when chlorine, used to disinfect drinking water, reacts with natural organic matter.

Our evaluation of NASEM sum is limited by including studies that did not measure all 7 of the NASEM analytes. Studies that included 6 of the 7 analytes may underestimate the Fluorine-Adjusted NASEM Sum. Three studies, Miyake et al., Yeung et al., and Pennoyer et al., did not measure MeFOSAA. An review of NHANES data from 2003–2004 detected MeFOSAA in 27.5% of samples, with the 95th percentile reporting an average of 1.3 ng/ml, equivalent to 0.74 ng F/ml using Eq. 1 [70].

There may also be limitations on using both the NASEM sum and PFAS burden scores in high exposed populations. The 2017–2018 US PFAS Burden Calculator is calibrated on data from the general population, using 2017–2018 PFAS biomonitoring data from the National Health and Nutrition Examination Survey. As such, in its current state, because it is calibrated on biomonitoring data from the general population, it may not be as informative in differentiating between highly exposed populations that have much higher PFAS exposure compared to that of the general population. Further work may be needed to enhance the PFAS burden calculator by calibrating on more diverse populations, and including additional sources of data that measure larger set of PFAS analytes. However, a key strength of the PFAS burden calculator is that the burden calculator can be used even if studies do not assay the same set of PFAS [47], while the NASEM sum requires studies to measure the full set of 7 analytes.

From our analysis, the small number of PFAS measured in the Adjusted NASEM Sum and the PFAS Burden Score explain a large portion of EOF in both the general population studies and the highly exposed community studies. This suggests that targeted approaches may provide reasonable rank-orders of total PFAS exposure. However, without additional and consistent measurements of additional organofluorines, including short-chain and non-persistent PFAS, fluorinated pharmaceuticals, and fluorinated pesticides, it is difficult to interpret whether the seven analytes consistently measured in these studies drive the correlations between the Adjusted NASEM Sum and the PFAS burden score. Further, the PFAS landscape could evolve, and the contribution of other organic-fluorinated chemicals (including pharmaceuticals and pesticides) may increase in the future.

Additional methods for estimating aggregate PFAS exposure are being developed and applied to biological matrices. Total oxidizable precursor assay (TOP assay) is an analytical technique which takes samples and converts PFAS precursors into perfluoroalkyl carboxylic acids (PFCAs) [71]. Similar to EOF, a strength of TOP assay is that it can detect PFAS without requiring a known chemical structure or standard. TOP assay is specific to PFCAs like EOF is specific to fluorine [72]. Particle-induced gamma ray emission (PIGE) spectroscopy is also being used as an approach that quantifies total fluorine on the surface of a sample [73]. PIGE and EOF differ in that EOF is a destructive approach where samples are combusted and destroyed, but samples are not combusted in PIGE, but exposed to radiation. EOF, TOP assay, and PIGE are all utilized in mass balance analyses to assess the gap between identifiable PFAS and total fluorine in a sample.

## Conclusions

In 8 studies, we found that serum EOF concentrations were correlated with both PFAS burden scores and NASEM sum. Overall, EOF concentrations were equal, or higher than NASEM summed PFAS concentrations. The amount of EOF explained by the Fluorine-Adjusted NASEM sum molecules was higher in populations with a known high PFAS exposure compared to populations with general exposure. As the PFAS landscape continues to evolve, targeted analysis of individual chemicals may not be sufficient or practical for estimating aggregate PFAS exposure. Comparison between summing targeted PFAS concentrations and EOF demonstrated that EOF is a practical tool for estimating PFAS exposure and accurately identifying individuals with high exposure to PFAS. Thus, EOF could be a useful tool in precision environmental health for identifying individuals or sub-populations with high aggregate PFAS exposure.

## Supplementary information

Supplementary information

## Data Availability

The datasets analyzed during the current study are available from the corresponding author on reasonable request.
