# Supplementary material for: Evaluation and comparison of tools used to quantify aggregate PFAS exposure: Extractable organic fluorine, PFAS burden scores and summed PFAS concentrations
Source: J Expo Sci Environ Epidemiol. 2025 Oct 2;35(6):1020–9. doi: 10.1038/s41370-025-00806-x (PMC12583196; doi:10.1038/s41370-025-00806-x)
Supplement: Supplementary file 1 — Supplementary information [file 41370_2025_806_MOESM1_ESM.pdf]

Supplementary Information for

Evaluation and Comparison of Tools Used to Quantify Aggregate PFAS Exposure: Extractable Organic Fluorine, PFAS Burden Scores and Summed PFAS Concentrations

Rachel Klein[a], Shelley H. Liu[b], Joseph M. Braun[c], Katherine E. Manz[a*]

[a]Department of Environmental Health Science, University of Michigan, 1415 E Washington Heights, Ann Arbor, MI, 48109, USA

[c]Department of Population Health Science and Policy, Icahn School of Medicine at Mount Sinai, New York, New York, 10029 USA

[c]Department of Epidemiology, Brown University, Providence, Rhode Island, 02912 USA

Keywords: Extractable organic fluorine, targeted PFAS analysis, PFAS burden scores, exposure assessment

*Address correspondence to Katherine E. Manz, M6242 SPHII, 1415 Washington Heights, Ann Arbor, MI 48103 USA. Email: katmanz@umich.edu

Supplemental Table 1a: Figure 1 Data Percentages

Study	MeFOSAA	PFDA	PFUnDA	PFHxS	PFNA	PFOA	PFOS	Non-NASEM F
A	0.040	0.818	0.022	2.092	0.782	2.566	13.071	80.609
B	0.039	1.015	0.605	4.229	1.558	4.335	30.415	57.803
C	0.218	1.077	1.706	3.305	1.945	5.998	52.367	33.385
D	0.146	3.218	1.669	6.319	5.439	26.795	24.069	32.345
E	0.000	0.678	0.816	11.763	1.886	11.414	64.764	8.679
F	0.000	0.622	0.577	2.124	0.732	1.446	60.499	34.000
G	0.000	0.838	0.434	16.481	3.220	10.562	39.404	29.062
H	0.276	0.417	0.433	5.375	0.614	4.368	23.652	64.864

Supplemental Table 1b: Figure 1b Data

Study	MeFOSAA	PFDA	PFUnDA	PFHxS	PFNA	PFOA	PFOS	Non-NASEM F
A	0.004	0.044	0.039	37.529	0.076	2.312	42.850	17.146
E	0.000	0.096	0.341	1.811	0.216	82.450	13.194	1.893

Supplemental Table 2a: Average EOF and Adjusted NASEM sum values for each study

Study	EOF (ng F/ml)	Adjusted NASEM Sum (ng F/ml)
A	24.9	4.82
B	8.88	3.75
C	20.7	13.8
D	2.65	2.38
E	35.2	23.2
F	25.6	13.8
G	6.50	4.80
H	41.4	12.9

Supplemental Table 2b:

Study	EOF (ng F/ml)	Adjusted NASEM Sum (ng F/ml)
A	234	206
E	755	754

Table 2c: Figure 2 Correlation Data

y~x	Exposure Status	Sample N	Spearman's r	P value	Figure Panel
EOF~Adjusted NASEM Sum	General Population	79	0.811	<0.01	A
EOF~Adjusted NASEM Sum	Known Exposure	23	0.858	<0.01	B

Table 3: Figure 3 Data

y~x	Exposure Status	Sample N	Spearman's r	P value	Figure Panel
EOF~PFAS Burden	General Population	79	0.721	<0.01	A
Adjusted NASEM Sum~PFAS Burden	General Population	139	0.883	<0.01	B
EOF~PFAS Burden	Known Exposure	23	0.403	0.056	C
Adjusted NASEM Sum~PFAS Burden	Known Exposure	24	0.541	0.0064	D
